# Protective Effects of *Lycium barbarum* Polysaccharide on 6-OHDA-Induced Apoptosis in PC12 Cells through the ROS-NO Pathway

**DOI:** 10.3390/molecules20010293

**Published:** 2014-12-24

**Authors:** Kai Gao, Meiyou Liu, Jinyi Cao, Minna Yao, Yunyang Lu, Jiankang Li, Xiaohe Zhu, Zhifu Yang, Aidong Wen

**Affiliations:** 1 Department of Pharmacy, Xijing Hospital, Fourth Military Medical University, Xi’an 710032, China; E-Mails: gaokai19881220@163.com (K.G.); lmy20140108@gmail.com (M.L.); caojinyi19@163.com (J.C.); yaona3698@163.com (M.Y.); 13720775293@163.com (J.L.); hexiaozhu1019@163.com (X.Z.); 2 Institute of Materia Medica, School of Pharmacy, Fourth Military Medical University, Xi’an 710032, China; E-Mail: luyunyanggq@163.com

**Keywords:** Parkinson’s disease, LBP, PC cells, apoptosis, reactive oxygen species, nitric oxide, mitochondrion

## Abstract

Oxidative stress plays an important role in Parkinson’s disease and other neurodegenerative disorders. *Lycium barbarum* polysaccharides (LBP), the main active ingredients extracted from the fruits of *Lycium barbarum* L., have been shown to be a potent antioxidant. In the present study, we investigated the protective effects, and the possible mechanism of action of LBP against 6-hydroxydopamine (6-OHDA)-induced apoptosis in PC12 cells. Our data demonstrated that LBP significantly reversed the 6-OHDA-induced decrease in cell viability, prevented 6-OHDA-induced changes in condensed nuclei and decreased the percentage of apoptotic cells in a dose-dependent manner. Furthermore, LBP also slowed the accumulation of reactive oxygen species (ROS) and nitric oxide (NO), decreased the level of protein-bound 3-nitrotyrosine (3-NT) and intracellular free Ca^2+^, and inhibiting the overexpression of nuclear factor κB (NF-κB), neuronal nitric oxide synthase (nNOS) and inducible nitric oxide synthase (iNOS). These results demonstrate that LBP prevents 6-OHDA-induced apoptosis in PC12 cells, at least in part through the ROS-NO pathway.

## 1. Introduction

Parkinson’s disease (PD), the second most common neurodegenerative disorder after Alzheimer’s disease (AD), is mainly characterized by pathological irreversible loss of dopaminergic (DAergic) neurons in the substantia nigra pars compacta (SNpc) [1,2]. Current PD medications mainly facilitate symptom management, but do not prevent disease progression [3]. The trigger for PD remains unknown, but the cascade of degenerative events leading to cell death is beginning to be understood. Current data indicates that oxidative damage represents a final common pathway in the pathogenesis of PD-like disorders [4,5,6], suggesting that compounds interfering with production of reactive oxygen species (ROS) and nitric oxide (NO) or with impairment of mitochondrial activity might be protective [7]. In addition, several lines of evidence in PD patients and animal models have suggested that oxygen-free radicals and oxidative stress are involved in the pathogenesis of PD [8,9,10]. Medicinal herbs that have antioxidative effects are now being considered as therapeutic agents against neuronal loss [7,11,12].

6-Hydroxydopamine (6-OHDA), a hydroxylated analog of the natural neurotransmitter, dopamine [13], can induce massive oxidative stress leading to the damage of DAergic neurons *in vitro* and *in vivo* [14,15,16]. PC12 cells, a cell line derived from rat adrenal pheochromocytoma cells, possess intracellular substrates for dopamine synthesis, metabolism and transport [14]. The apoptosis of PC12 cells induced by 6-OHDA has been used as an *in vitro* experimental model for the study of PD [17,18].

The fruits of *Lycium barbarum* L. (family Solanaceae), commonly known as Goji berry or wolfberry, have been a famous traditional Chinese herbal medicine for thousands of years. *Lycium barbarum* polysaccharide (LBP), which is the major active component of the fruits, has been found to have several biological activities, including antioxidative activity, immunomodulation, anti-cancer, anti-aging activity [19,20,21] and neuroprotective properties. For example, LBP has been reported to have neuroprotective effect against the cerebral reperfusion-induced injury in the brain through reducing lipid peroxides, scavenging free radicals, and improving the energy metabolism [22]. Moreover LBP has been shown to be a new PI3K/AKT/Nrf2 axis activator, prevented the development of oxidative stress [23]. In the preliminary study, we confirmed that LBP had neuroprotective effects related to treatment of PD, but the protective effects of LBP on 6-OHDA-induced apoptosis in PC12 cells remain unknown.

Therefore, the present study was designed to verify the potential neuroprotective effects of LBP against 6-OHDA-inducedapoptosis in PC12 cells and the possible mechanisms by measuring the ratio of apoptotic cells, intracellular ROS, intracellular nitric oxide (NO), intracellular free Ca^2+^, protein levels of nuclear factor κB (NF-κB), inducible nitric oxide synthase (iNOS) and neuronal nitric oxide synthase (nNOS), and the level of 3-nitrotyrosine (3-NT).

## 2. Results and Discussion

### 2.1. Results

#### 2.1.1. LBP Prevents 6-OHDA -Induced Apoptosis of pc12cells

**Figure 1:**
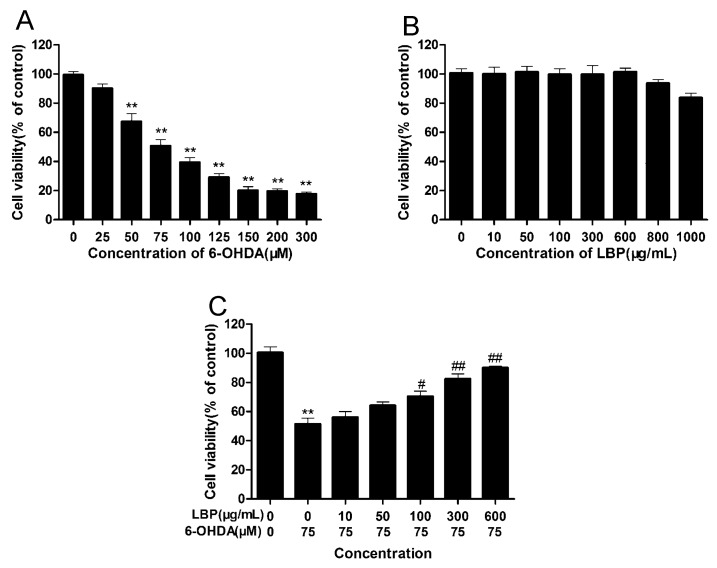
Effects of LBP and 6-OHDA on cell viability. Cells were incubated for 24 h in different concentrations of 6-OHDA alone (**A**) or indifferent concentrations of LBP alone (**B**); Cells were preincubated with different concentrations of LBP (**C**) for 24 h, and then exposed to 6-OHDA (75 μM) for 24 h. Data are expressed as percentage of the untreated control ± SD (*n* = 3). ** *p* < 0.01 compared with untreated control cells; ^#^ *p* < 0.05, ^##^ *p* < 0.01 compared with 6-OHDA-treated cells.

After incubation with various concentrations of 6-OHDA for different periods of time, the viability of PC12 cells was determined using the MTT assay. There was a dose- and time-dependent decrease in cell viability following 6-OHDA exposure (Figure 1A). After incubation with 75 μM of 6-OHDA for 24 h, only 50% of cultured cells survived, and this concentration was used in the following experiments. To determine whether LBP alone had any effects on cell viability, PC12 cells treated with various concentrations of LBP for 24 h. Results showed that LBP alone had no obvious effect on cell viability (Figure 1B). Conversely, cells treated with various concentrations of LBP for 24 h before the addition of 6-OHDA (75 μM) for 24 h showed that cell viability increased at concentrations of LBP (100–600 μg/mL) (Figure 1C).

This suggested that LBP could effectively protect PC12 cells against 6-OHDA-induced cell death. And, we also found LBP protected primary neurons from 6-OHDA induced cell death (Figure S1A).

#### 2.1.2. LBP Rescues 6-OHDA -Induced Changes in Nuclear Morphology

**Figure 2:**
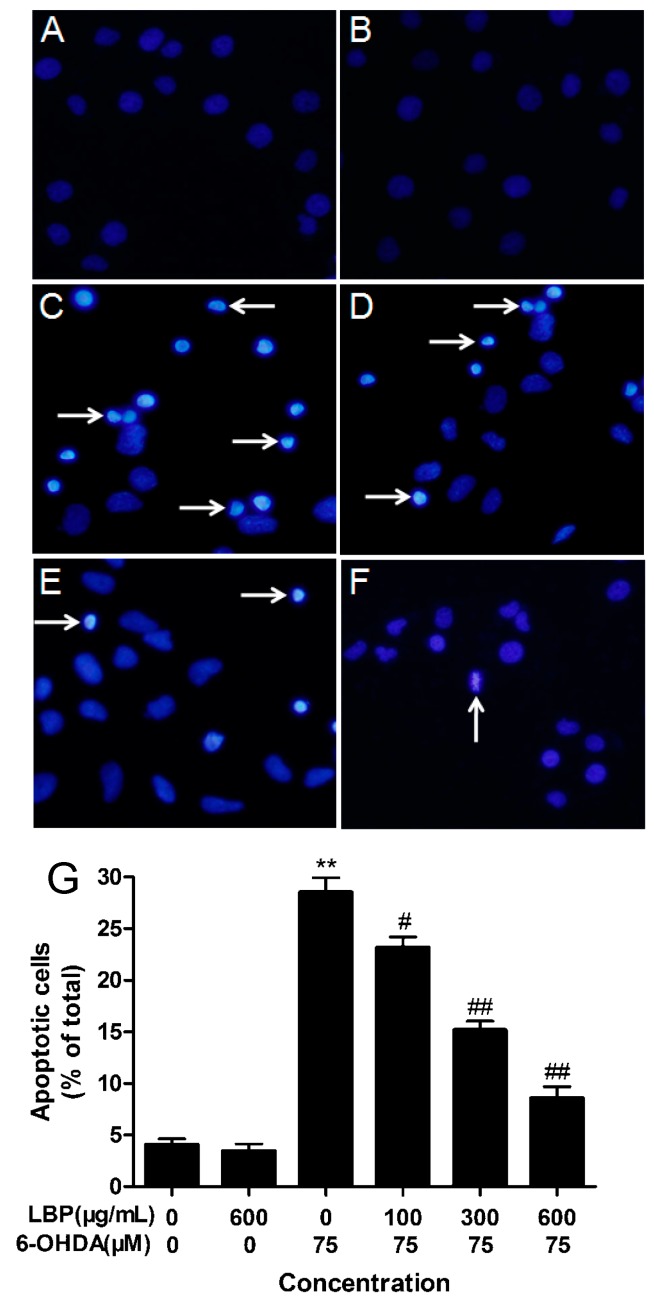
LBP rescues 6-OHDA-induced changes in nuclear morphology. Nuclear morphology was assessed using DAPI staining. (**A**) shows normal culture medium nucleic morphology, (**B**,**C**) respectively show cells cultured in 600 μg/mL LBP or 75 μM 6-OHDA for 24 h. In addition, cells were pretreated with 100 μg/mL (**D**), 300 μg/mL (**E**) or 600 μg/mL (**F**) LBP for 24 h and then incubated in 6-OHDA (75 μM) for an additional 24 h. (**G**) Histograms showing ratio of condensed nuclei to total nuclei. (** *p* < 0.01 compared with untreated control cells; ^#^ *p* < 0.05, ^##^ *p* < 0.01 compared with 6-OHDA-treated cells). White arrows represent location of apoptosis cell. Scale bars represent 50 mm.

Nuclear morphology was assessed using DAPI staining. The normal nucleus showed a homogeneous staining, bearing regular contours and rounded shapes (Figure 2A). Apoptotic nuclei indicated by condensed nuclei and nuclear fragmentation were apparent after exposure to 75 μM 6-OHDA (Figure 2C).

These changes in nuclear characteristics of apoptosis were rescued significantly in the cells pretreated with the different concentrations of LBP (100–600 μg/mL) (Figure 2D–F). However, LBP alone had no effect (Figure 2B).

#### 2.1.3. LBP Rescues 6-OHDA -Induced Apoptosis

**Figure 3:**
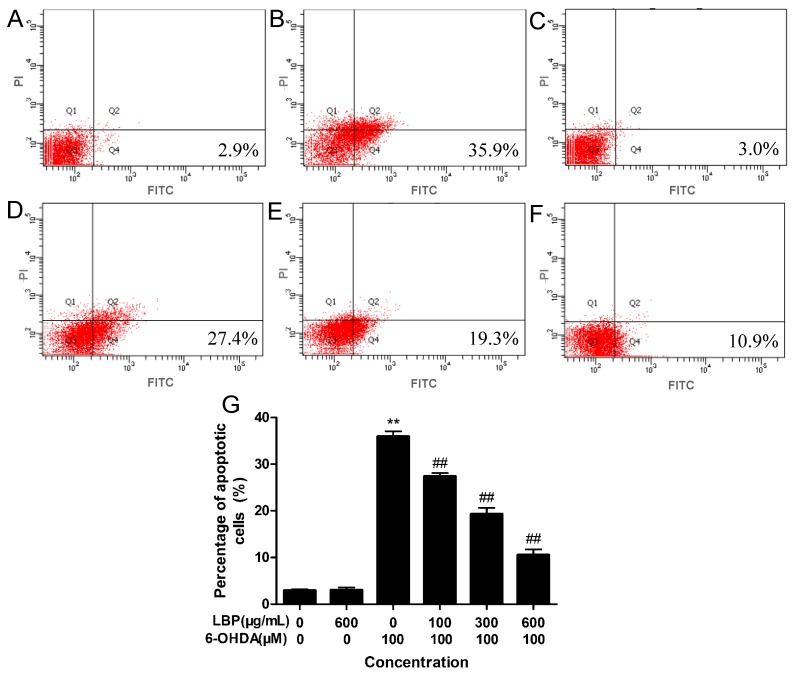
Cell apoptosis was tested by flow cytometry analysis. PC12 cells were incubated in drug-free medium (**A**) or medium containing 600 μg/mL LBP; (**B**) or 75 μM 6-OHDA; (**C**) for 24 h, or cells were preincubated with 100 μg/mL (**D**); 300 μg/mL (**E**) or 600 μg/mL (**F**) of LBP. The results shown in (**G**) are the means ± SD for three independent experiments. ** *p* < 0.01 compared with untreated control cells; ^##^ *p* < 0.01 compared with 6-OHDA-treated cells.

The annexin-V^−^/PI^−^ population is made up of normal healthy cells, while annexin-V^+^/PI^−^ cells exist in early apoptotic stage, and annexin-V^+^/PI^+^cells exist in late apoptotic/necrotic stage. Treatment with 75 μM 6-OHDA increased the percentage of apoptotic cells (35.9%) (Figure 3C) compared to the control group (2.9%) (Figure 3A). Pretreatment with LBP (100–600 μg/mL) before treatment with 6-OHDA reduced the percentage of apoptotic cells to 27.4%, 19.3% and 10.9%, respectively (Figure 3D–F). LBP alone did not display any obvious effect (Figure 3B).

#### 2.1.4. Effects of LBP on Intracellular ROS Levels

**Figure 4:**
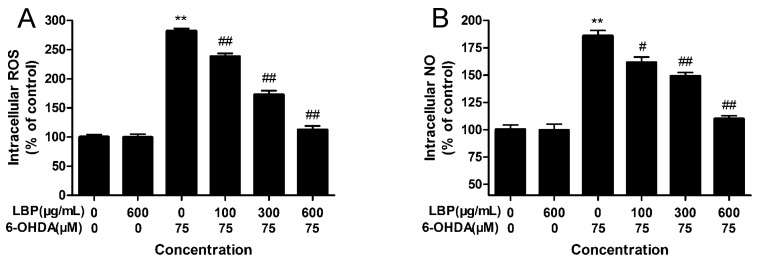
Effect of LBP on 6-OHDA-induced accumulation of intracellular ROS (**A**) and NO (**B**) levels. Cells were exposed to 6-OHDA without or with different concentrations of LBP for 24 h. Data are expressed as percentage of untreated control cells ± SD (*n* = 6). ** *p* < 0.01 compared with untreated control cells; ^#^ *p* < 0.05, ^##^ *p* < 0.01 compared with 6-OHDA-treated cells.

The level of intracellular ROS was examined by using DCFH-DA. As shown in Figure 4A, treatment of PC12 cells with 75 μM 6-OHDA for 24 h led to an increase in DCF fuorescence with an obvious increment than control (*p* < 0.01). However, this increase in DCF fluorescence was decreased in a concentration-dependent manner by pretreatment with LBP (100–600 μg/mL). Additionally, LBP alone had no obvious effect on intracellular ROS levels. In primary neurons, LBP effectively inhibited accumulation of intracellular ROS (Figure S2A).

#### 2.1.5. Effects of LBP on Intracellular NO Levels

Intracellular NO levels were measured with DAF-FM DA. Exposure of PC12 cells to75 μM 6-OHDA for 24 h led to a rapid increase in DAF-FM fluorescence, compared with the control group (*p* < 0.01) (Figure 4B). LBP (100–600 μg/mL) pretreatment inhibited such an increase in DAF-FM fluorescence, while LBP alone had no effect on DAF-FM fluorescence intensity (Figure 4B). In primary neurons, LBP effectively inhibited accumulation of intracellular NO (Figure S2B).

#### 2.1.6. LBP Inhibits 6-OHDA -Induced Elevation in [Ca^2+^]_i_

**Figure 5:**
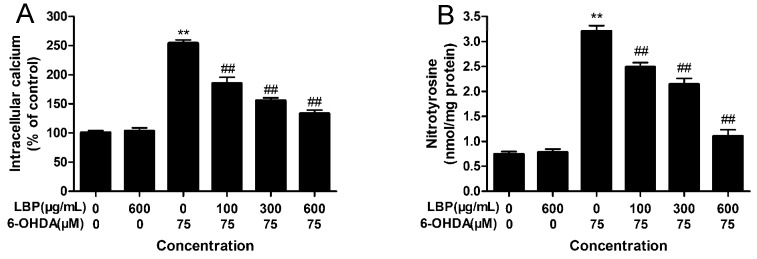
LBP attenuates the 6-OHDA-induced elevation of intracellular [Ca^2+^]_i_ (**A**) and protein-bound 3-NT (**B**) in PC12 cells.PC12 cells were exposed to LBP (100–600 μg/mL) for 24 h before 75 μM 6-OHDA was added to the medium for an additional 24 h. Data are expressed as percentage of untreated control cells ± SD (*n* = 6). ** *p* < 0.01 compared with untreated control cells; ^##^ *p* < 0.01 compared with 6-OHDA-treated cells.

To examine the intracellular calcium concentration in apoptotic cells, PC12 cells were incubated with Fluo-3AM. As shown in Figure 5A, exposure of cells to 6-OHDA (75 μM) resulted in an obvious elevation of [Ca^2+^]_i_ (*p* < 0.01); Treatment with LBP alone had no obvious influence on the level of [Ca^2+^]_i_; Pretreatment with LBP (100–600 μg/mL) for 24 h before treatment with 6-OHDA for another 24 h provoked a significant decrease in [Ca^2+^]_i_, compared with the 6-OHDA-treated cells (*p* < 0.01). These suggest that the protective effect of LBP on the cells is against the elevation of [Ca^2+^]_i_ caused by 6-OHDA.

#### 2.1.7. LBP Inhibits the 6-OHDA -Induced Elevation in Protein-Bound 3-NT

Peroxynitrite nitrates protein-bound tyrosine to produce3-NT. Protein-bound 3-NT was measured by a competitive ELISA method with an anti-3-NT antibody. Figure 5B shows that 6-OHDA increases the level of protein-bound 3-NTcompared with the control group (*p* < 0.01), and LBP pretreated cells showed a reverse effect in a dose-dependent manner in the 100–600 μg/mL ranges, compared with the 6-OHDA group. LBP alone had no effect on the protein-bound 3-NT level in PC12 cells.

#### 2.1.8. Effects of LBP on the Expression of nNOS and iNOS

**Figure 6:**
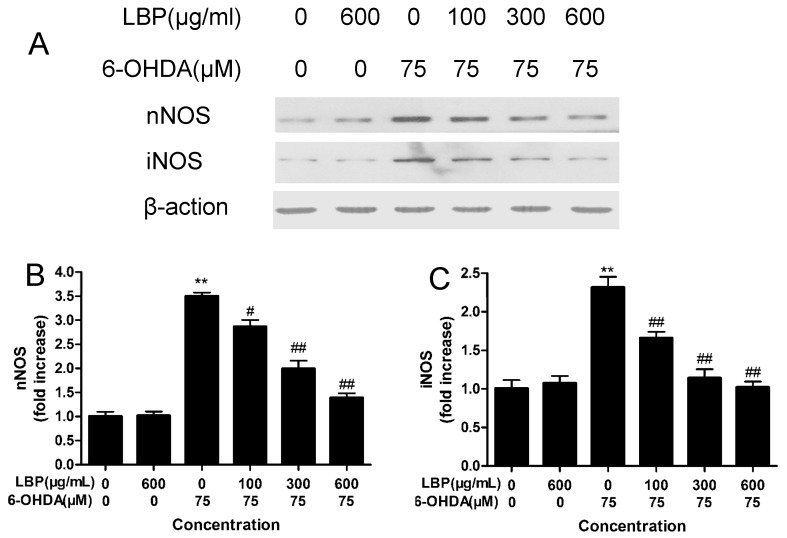
Effect of LBP and 6-OHDA on the expression of nNOS and iNOS. PC12 cells were exposed to 6-OHDA (75 μM) with or without various concentrations of LBP for 24 h, and the nNOS and iNOS were detected by western blot (**A**); The protein levels and date analysis for nNOS (**B**) and iNOS (**C**); Data obtained from quantitative densitometry were presented as mean ± SD for three independent experiments. ** *p* < 0.01 compared with untreated control cells; ^#^ *p* < 0.05, ^##^ *p* < 0.01 compared with 6-OHDA-treated cells.

6-OHDA exposure induced up-regulation of nNOS and iNOS as revealed by western blot analysis. LBP pre-treatment reduced the expression of nNOS and iNOS induced by 6-OHDA in a dose-dependent manner (Figure 6). Treatment with LBP alone had no obvious influence on the expression of iNOS and nNOS in PC12 cells (Figure 6). In addition, LBP pre-treatment could also reduce the expression of NF-κB induced by 6-OHDA in a dose-dependent manner (Figure S3A,C).

#### 2.1.9. Effects of LBP on Caspase 3 and Caspase 9 Activities

The mitochondria-mediated apoptosis involves the activation of caspase 3 as well as caspase 9. The activities of caspase 3 and caspase 9 were measured by western blotting analysis. Our results shown in Figure S3B, D and E indicated that LBP could attenuate the increase in cleaved caspase-3 level induced by 6-OHDA in a dose-dependent manner.

### 2.2. Discussion

The present study shows that treating PC12 cells with 6-OHDA significantly reduced cell viability, induced typical apoptosis features, increased the level of intracellular ROS and NO, elevated the level of 3-NT and intracellular Ca^2+^, and induced overexpression of iNOS and nNOS. However, the above changes were markedly reversed in a dose-dependent manner after the pretreatment of PC12 cells with different concentrations of LBP for 24 h, which suggests that LBP may protect PC12 cells from 6-OHDA-induced apoptosis by regulating the ROS-NO pathway.

Oxidative stress has been strongly implicated in neurodegeneration associated with PD. 6-OHDA is a neurotoxin that produces oxidative stress in both *in vitro* and *in vivo* experimental PD models [24]. Studies have reported that the toxic effects of 6-OHDA result from the overproduction of ROS through three pathways: extracellular auto-oxidation, intracellular metabolism by monoamine oxidase and direct inhibition of mitochondrial respiratory chain [25,26]. Moreover, the generation of intracellular ROS by 6-OHDA is an initial event and the ROS suppresses the Akt phosphorylation, increases p38 phosphorylation which induces the activation of caspase-9 as well as caspase-3, and finally leads to cell apoptosis [26]. These indicate that ROS plays an important role in cell apoptosis induced by 6-OHDA. In addition, NO is a well-known vasorelaxant agent, while NO produced by neurons plays an important role as a neurotransmitter and NO produced by immune and glial cells is involved in defense functions [27]. Postmortem pathological studies in brains of patients with PD also have suggested that NO plays an important role in PD [28,29]. In the present study, we found that cells treated with 6-OHDA for 24 h show an increase in levels of ROS and NO. However, Pretreatment with LBP prior to 6-OHDA treatment provided a dose-dependent counteraction of these changes, suggesting that ROS and NO reduction are characteristic of the neuroprotection accorded by LBP.

NO and its toxic metabolite peroxynitrite (ONOO^−^) can inhibit or damage the mitochondrial complexes I, II, IV and V, aconitasa, creatin-quinase, mitochondrial membrane, mitochondrial DNA and mitochondrial SOD, and induces Ca^2+^ release, transient permeability, cytochrome c release and mitochondrial swelling [30]. Excessive mitochondrial Ca^2+^ accumulation may impair mitochondrial function and activate certain phospholipases, proteases and endonucleases, leading to irreversible membrane, organelle and chromatin damage and eventually to cell death [31]. The cytotoxic effects of NO and ONOO^−^ are the consequence of DNA damage leading to p53 accumulation and consequently to p21 upregulation [27]. Moreover, (ONOO^−^) could contribute to the depletion of major cellular antioxidant defense, making the nigrostriatal pathway especially susceptible to toxic insult, and has been implicated in the apoptosis of dopaminergic neurons in PD [32,33]. It is hard to detect ONOO^−^, thus, the generation of ONOO^−^ is usually measured by levels of 3-NT, a footprint of ONOO^−^. Our results show that 6-OHDA elevated the level of intracellular Ca^2+^ and 3-NT in PC12 cells, while the level of intracellular Ca^2+^ and 3-NT decreased after pretreatment of PC12 cells with LBP.

NO is synthesized from L-arginine by NOS in the presence of reduced nicotinamide adenine dinucleotide phosphate and molecular oxygen [34]. To date, NOS has four known isoforms: neuronal NOS (nNOS), endothelial NOS (eNOS), inducible NOS (iNOS) and mitochondrial NOS (mtNOS). The last one is an isoform of nNOS present in the inner mitochondrial membrane [27]. nNOS and iNOS are acknowledged to be closely related to the pathogenesis of PD [35]. In nNOS knockout mice, 1-methyl-4-phenylpyridinium (MPP^+^) or 1-methyl-4-phenyl-1,2,5,6-tetrahydropyridine (MPTP)-induced neuronal damage is reduced compared with wild type mice [36]. In another experimental model of PD, iNOS was reported to be involved in the induction neuronal damage by 6-OHDA [37]. nNOS inhibitor has a dose-dependent protective effect against MPTP-induced striatal dopamine and 3,4-dihydroxyphenylacetic acid depletion in mice [38]. However, the ROS-induced disruption of Ca^2+^ homeostasis could enhance nNOS without requiring external activation. The expression and activity of iNOS are dependent on the activity of NF-κB [7]. In our present study, western blot analyses showed that the level of intracellular Ca^2+^, and the expression of NF-κB, nNOS and iNOS proteins increased after treatment of PC12 cells with 6-OHDA for 24 h. Conversely, the level of intracellular Ca^2+^, the activation of NF-κB (Figure S3A,C), and expression of nNOS and iNOS decreased in a dose-dependent manner after pretreatment with different concentrations of LBP in PC12 cells. It is suggested that LBP protect against 6-OHDA-induced cells apoptosis through blocking nNOS and iNOS via down-regulation of intracellular Ca^2+^ and NF-κB pathway. In addition, we also found that inhibition of NOS generation by L-NMMA (an NOS inhibitor) can partially counteract 6-OHDA-induced apoptosis. These data suggest that NO plays an important role in the apoptosis induced by 6-OHDA, and LBP may decrease the level of cell apoptosis induced by 6-OHDA.

## 3. Experimental Section

### 3.1. Preparation of Lycium Barbarum Polysaccharide

LBP was isolated from the fruits of *L. barbarum* by water extraction and ethanol precipitation, followed by removal of lipids and oligosaccharides. Briefly, the dried fruits of *L. barbarum* were refluxed to remove lipids with chloroform: methanol solvent (2:1) (v/v). After filtering, the residues were air-dried, and then refluxed again with 85% ethanol at 80 °C to remove oligosaccharides. The residues were put in boiling deionized water. The water extract was filtered through a filter paper to remove impurities. Then the extract was concentrated by a rotavapor at 60 °C, and then precipitated using 95% ethanol. The precipitate was washed in turn with 100% ethanol, 100% Ether and acetone. After filtering and centrifuging, the precipitate was collected and vacuum-dried. The dried *L. barbarum* polysaccharides (LBP) obtained were dissolved in PBS or normal saline, filtered through a 0.22-um filter, and stored at 4°C.

### 3.2. Reagents

6-Hydroxydopamine hydrochloride (6-OHDA), 3-(4,5-dimethylthiazol-2-yl)-2,5-diphenyltetra-zolium bromide (MTT) were purchased from Sigma (St. Louis, MO, USA). Dulbecco’s modified Eagle’s medium (DMEM), fetal calf serum (FCS) and horse serum were purchased from Gibco (Gaithersburg, MD, USA). Rabbit polyclonal antibodies to nNOS and to iNOS were purchased from Abcam Company (Cambridge, UK). A 3-NTELISA kit was purchased from the Xitang Institute of Biotechnology (Shanghai, China). 3-Amino-4-aminomethyl-2',7'-difluorescein diacetate (DAF-FMDA), Reactive Oxygen Species Assay Kit, and donkey anti-goat lgG-HRP were purchased from the Beyotime Institute of Biotechnology (Shanghai, China).

### 3.3. Primary Culture of Rat Cortical Neurons

Cortical neurons were prepared from brains of one-day-old Sprague-Dawley rats. Approximately 30,000 cells in 50 mL neurobasal medium containing glutamine (1 mmol/L), 1% penicillin, streptomycin (Pen/Strep), and 10% fetal bovine serum were seeded into 6-well plates. After 2 h, 0.5 mL neurobasal medium containing the serum-free B27 supplement (2%), Pen/Strep, and glutamine were added to each well. After 2 days *in vitro*, 5 mM cytosine arabinofuranoside was added to inhibit neuronal proliferation. At 5 days *in vitro*, the medium was changed to fresh neurobasal medium containing B27. Neurons were cultured at 37 °C in a humidified 5% CO_2_ atmosphere and used after 7 days *in vitro*.

### 3.4. PC12 Cell Culture and Treatment

PC12 cells were cultured in DMEM supplemented with 10% heat-inactivated horse serum, 5% heat-inactivated FCS, 100 IU/mL penicillin and 100 μg/mL streptomycin. The cultures were maintained in a 5% CO_2_/95% air humidified atmosphere at 37 °C, and the culture medium was changed every 2–3 days. PC12 cells were differentiated for up to 9 days in the culture medium supplemented with 50 ng/mL NGF [39]. Cells were seeded on poly-l-lysine-coated plates and passaged at 60%–70% confluence. In all experiments, other than assessments of cell viability, cells were treated with varying concentrations of 6-OHDA (25, 50, 75, 100, 125, 150, 200, 300 μM) for 24 h to investigate the neurotoxicity of 6-OHDA. LBP was dissolved in distilled water and diluted with cell culture medium. Cells were treated with 100–600 μg/mL LBP for 24 h, washed three times with phosphate-buffered saline (PBS), and then incubated with 75 μM 6-OHDA for an additional 24 h. In the investigation, the protective mechanism of LBP against 6-OHDA-induced cells apoptosis, PC12 cells were divided into control group, LBP treatment group alone, 6-OHDA treatment group alone, and a group pretreated with different concentrations of LBP (100–600 μg/mL) followed by 6-OHDA treatment.

### 3.5. Assessment of Cell Viability

Cell viability was measured by MTT assay, which is based on the conversion of MTT to formazan crystals by mitochondrial dehydrogenases [40]. PC12 cells (1.0 × 10^4^ cells/well) were seeded into 96-well plates. After pretreatment with four fractions for 2 h, the cells were incubated with 6-OHDA for 24 h, the cells were incubated with 20 μL of MTT solution (5 mg/mL in PBS) for 4 h at 37 °C. The medium was carefully removed, and 150 μL of DMSO was added. The absorbance was determined at 570 nm using a microplate reader (Bio-Rad, Hercules, CA, USA). Control cells were treated in the same way without 6-OHDA treatment, and the values of different absorbances were expressed as a percentage of the control.

### 3.6. Morphological Changes

The changes in nuclear morphology of apoptotic cells were detected using the DNA-specific fluorescent dye 4'-6-diamidino-2-phenylindole (DAPI) (Invitrogen, Carlsbad, CA, USA) staining [41]. After being treated with 6-OHDA and/or LBP for 24 h, the cells were fixed with 3% paraformaldehyde for 30 min at room temperature, then washed twice with PBS. DAPI was added to the fixed cells for 5 min, after which they were examined by fluorescence microscopy (Nikon, Tokyo, Japan) to assess chromatin condensation and fragmentation of nuclei. Cells that exhibited reduced nuclear size, chromatin condensation, intense fluorescence and nuclear fragmentation were considered as apoptotic. Cells were counted in five randomly chosen slides and the number of apoptotic cells is expressed as a percentage to total cells counted.

### 3.7. Flow Cytometric Analysis

Apoptotic and necrotic cells were quantified using Annexin V binding and PI uptake [42]. Briefly, after treatment with 6-OHDA and/or LBP, cells were harvested by centrifugation, washed with ice-cold PBS, and resuspended in 100 μL binding buffer. A total of 5 μL of 20 μg/mL Annexin V and 50 μg/mL PI were added, and the tube incubated for 15 min at room temperature in the dark. Quantitative analysis of the level of apoptosis was performed using a flow cytometer (BD Biosciences, Franklin, NJ, USA). Apoptotic cells were expressed as a percentage of the total number of cells.

### 3.8. Measurement of Intracellular ROS

Intracellular ROS was monitored by using the 2',7'-dichlorofuorescin diacetate (DCFH-DA) fuorescent probe [43]. Briefly, after PC12 cells were treated with 6-OHDA and/or LBP for 24 h, cells were incubated with 10 μM DCFH-DA at 37 °C for 30 min, and then washed twice with PBS. Finally the fluorescence intensity of DCF was measured by a multi-detection microplate reader with excitation at 488 nm and emission at 530 nm within 15 min. The measured fluorescence values were expressed as a percentage of the fluorescence in control cells.

### 3.9. Measurement of Intracellular NO

The intracellular NO was detected using 3-amino,4-aminomethyl-2',7'-difluorescein diacetate (DAF-FM DA), a nitric oxide fluorescent probe, reacts with NO in viable cells to produce a fluorescent compound [44]. Briefly, after PC12 cells were treated with 6-OHDA and/or LBP for 24 h, the cells were then incubated with 5 μM DAF-FM DA for 20 min at 37 °C. After the cells were washed with PBS, the fluorescence intensity was analyzed by a multi-detection microplate reader with excitation at 495 nm and emission at 515 nm within 15 min. The measured fluorescence values were expressed as a percentage of the fluorescence in control cells.

### 3.10. LBP Inhibits 6-OHDA -Induced Elevation in [Ca^2+^]_i_

The concentration of intracellular Ca^2+^ was measured with Fluo-3 AM by the method of Guo *et al.* [7] with modification. PC12 cells were harvested after 6-OHDA or LBP incubation, washed, and resuspended in a serum-free medium, and then Fluo-3 AM (5 mM) was added, which was subsequently incubated for 30 min at 37 °C. After washing three times, cells were resuspended in the standard medium and transferred to a fluorometer cuvette. The fluorescence intensity of Fluo-3 was quantified by a fluorescence spectrophotometer at an excitation wavelength of 490 nm and an emission wave-length of 520 nm. [Ca^2+^]_i_ was calculated from the Fluo-3fluorescence intensity using the equation:

(1) $${\left[{\text{Ca}}^{2+}\right]}_{\text{i}}=K_{d}(F-F_{min})/(F_{max}-F)$$

For the purpose of calculation of [Ca^2+^]_i_, *K*_d_ as dissociation constant is 400 nM. The maximal Fluo-3 fluorescence intensity (*F*_max_) was determined by adding 0.1% Triton X-100 and the minimal fluorescence (*F*_min_) was determined by quenching Fluo-3fluorescence with 5 mM EGTA. *F* is the fluorescence measured without adding Triton X-100 or EGTA.

### 3.11. Measurement of Protein-Bound 3-NT

Protein-bound3-NT was detected with the ELISA method according to the manual [45]. Briefly, a nitrated protein solution was prepared and diluted for use as a standard. These standard samples and cell samples were pipetted into 96-well plates and incubated with a rabbit polyclonal anti-nitrotyrosine primary antibody for1 h at 37 °C. Then, samples were incubated with a horseradish peroxidase-conjugated secondary antibody for 1 h and washed. Subsequently, these samples were incubated with freshly prepared LumiGLO Chemiluminescent Substrate for 10 min. Luminescence was then measured with a microplate reader. The nitrotyrosine content in cell samples was calculated by standard curves generated from nitrated bovine serum albumin containing quantified nitrotyrosine amounts.

### 3.12. Western Blot Analysis of nNOS and iNOS

After treatment by 6-OHDA and/or LBP, PC12 cells were lysed by lysis buffer (50 mM Tris-Cl, 150 mM NaCl, 0.02% (w/v) NaN_2_, 100 μg/mL PMSF, 1 μg/mL aprotinin, and 1% (v/v) Triton X-100). The lysate was incubated on ice for 30 min and then centrifuged at 12,000 *g* for 5 min at 4 °C. The supernatant was collected to use for SDS-PAGE and the protein content was estimated by the method of Bradford [46]. Total proteins were separated on 8% polyacrylamide gels and transferred to a nitrocellulose membrane. The membrane was incubated in a fresh blocking buffer (5% (v/v) bovine serum, 0.1% (v/v) Tween 20 in 0.1 MPBS, pH 7.4) at room temperature for 30 min, then incubated with primary antibody (diluted 1:1000) overnight at 4 °C, Following three washes with PBS/Tween 20, the membranes were incubated with horseradish peroxidase (HRP)-conjugated donkey anti-goat IgG antibody (1:2000) at room temperature for 1 h. The membrane was washed and stained with 3,3'-diaminobenzidine tetrahydrochloride (DAB). After washing, blotted proteins were visualized using a western blotting detection system (ECL Plus, Amersham, Buckinghamshire, UK) and quantified with NIH Image software (National Institutes of Health, Bethesda, MD, USA).

### 3.13. Statistical Analysis

All experiments were performed at least three times. Data are expressed as means ± SD and were analyzed statistically by one-way ANOVA analysis of variance with subsequent Bartlett’s test. Differences where *p* < 0.05 were considered statistically significant.

## 4. Conclusions

In summary, our data indicate that LBP inhibits the apoptosis induced by 6-OHDA in PC12 cells. The protective effects of LBP are related to slowing the accumulation of intracellular ROS and NO, decreasing the levels of 3-nitrotyrosine (3-NT) and intracellular free Ca^2+^, and inhibiting the overexpression of NF-κB, nNOS and iNOS. Those results show that the protective effects of LBP on PC12 cells are mediated, at least in part, by regulating the ROS–NO pathway. Therefore, LBP acting as a powerful antioxidant, may become a neuroprotective agent in the treatment of neurodegenerative diseases, and should next be further studied on the mechanism of neuroprotective effect.

## Supplementary Materials

Supplementary data associated with this article can be found, in the online version, at http://www.mdpi.com/1420-3049/20/01/0293/s1.
