# Supplementary material for: Protective Effects of Lycium barbarum Polysaccharide on 6-OHDA-Induced Apoptosis in PC12 Cells through the ROS-NO Pathway
Source: Molecules. 2014 Dec 24;20(1):293–308. doi: 10.3390/molecules20010293 (PMC6272587; doi:10.3390/molecules20010293)
Supplement: Supplementary file 1 [file molecules-20-00293-s001.pdf]

Supplementary Materials

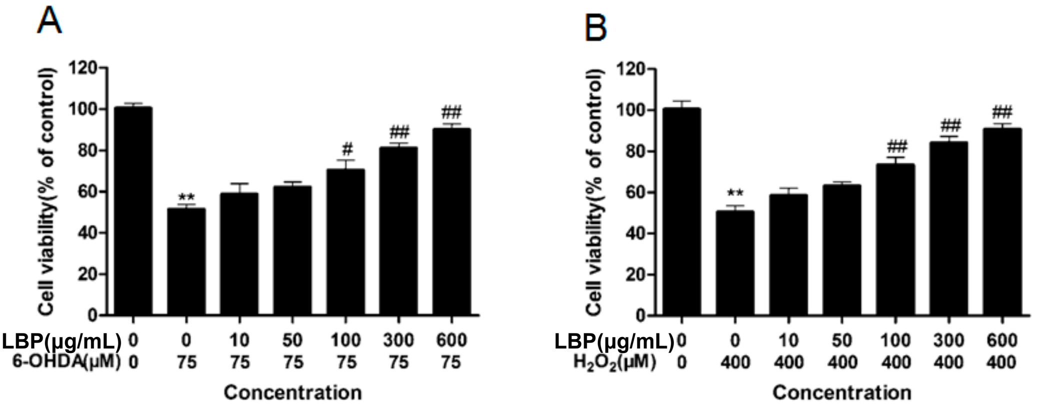

Figure S1. Cell viability was determined by MTT assay. Primary cells were preincubated with different concentrations of LBP (**A**) for 24 h, and then exposed to 6-OHDA (75 μM) for 24 h. Data are expressed as percentage of untreated control cells ± SD (n = 3). ** $p < 0.01$ compared with untreated control cells; # $p < 0.05$, ## $p < 0.01$ compared with 6-OHDA-treated cells. PC12 cells were preincubated with different concentrations of LBP (**B**) for 24 h, and then exposed to H_2O_2 (400 μM) for 24 h. Data are expressed as percentage of untreated control cells ± SD (n = 3). ** $p < 0.01$ compared with untreated control cells; # $p < 0.05$, ## $p < 0.01$ compared with H_2O_2-treated cells.

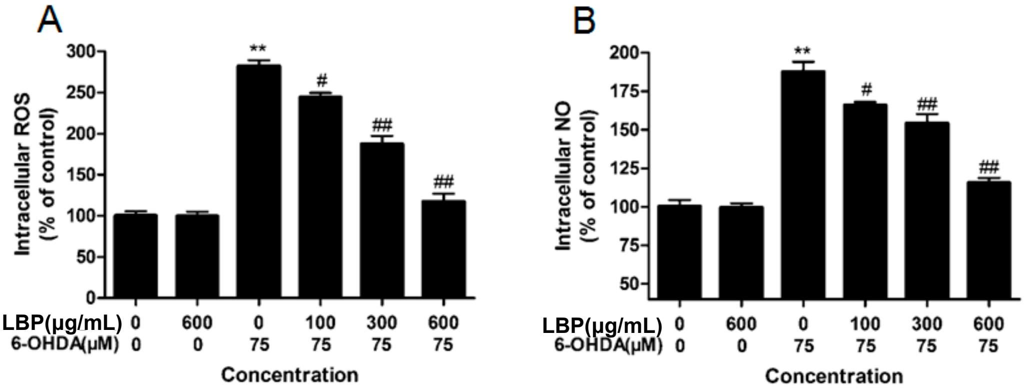

Figure S2. Effect of LBP on 6-OHDA-induced accumulation of intracellular ROS (**A**) and NO (**B**) levels in primary cells. Cells were exposed to 6-OHDA without or with different concentrations of LBP for 24 h. Data are expressed as percentage of untreated control cells ± SD (n = 6). ** $p < 0.01$ compared with untreated control cells; # $p < 0.05$, ## $p < 0.01$ compared with 6-OHDA-treated cells.

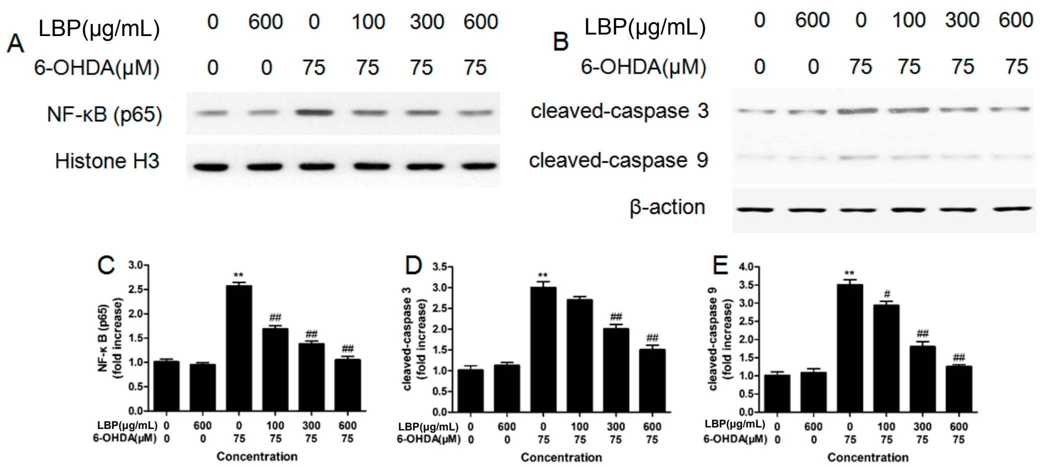

Figure S3. Effects of LBP on the activation of NF-κB, Caspase 3 and Caspase 9. PC12 cells were exposed to 6-OHDA (75 μM) with or without various concentrations of LBP for 24 h. The changes in expression of NF-κB in the nuclear extractions were examined by Western blot analysis (**A**,**B**). NF-κB (p65), caspase 3 and caspase 9 activities were measured (**C**–**E**). Data obtained from quantitative densitometry were presented as mean ± SD for three independent experiments. ** $p < 0.01$ compared with untreated control cells; $^{\#}$ $p < 0.05$, $^{\#\#}$ $p < 0.01$ compared with 6-OHDA-treated cells.

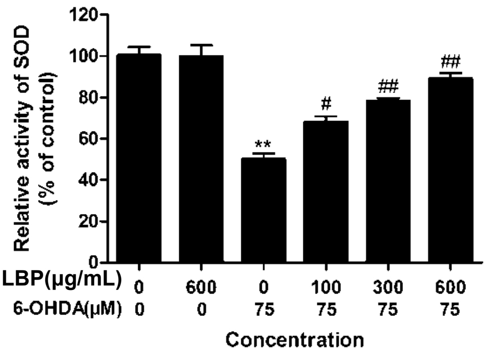

Figure S4. Effect of LBP on enzymatic activity of SOD in 6-OHDA-treated cells. PC12 cells were pretreated with different concentrations of LBP, and then exposed to 75 μM 6-OHDA for 24 h. Data are expressed as percentage of untreated control cells ± SD ($n = 6$). ** $p < 0.01$ compared with untreated control cells; $^{\#}$ $p < 0.05$, $^{\#\#}$ $p < 0.01$ compared with 6-OHDA-treated cells.
